# Mitigating effect of naringenin on potassium bromate-induced nephrotoxicity in vivo

**DOI:** 10.1007/s00210-026-05271-3

**Published:** 2026-04-22

**Authors:** Iftekhar Hassan, Hossam Ebaid, Badar ul Islam, Jameel Al-Tamimi, Zeinab Abdelftah, Shazia Aman, Ibrahim M. Alhazza, Ezzat M. Awad

**Affiliations:** 1 Department of Zoology, College of Science, King Saud University, P.O. Box 2455, Riyadh, 11451 Saudi Arabia; 2 Department of Biochemistry, Ram Gopal Medical College and Research Center, Hathras, India; 3 Comparative Anatomy and Embryology Division, Zoology Department, Faculty of Science, Beni-Suef University, Beni-Suef, 62521 Egypt; 4 Department of Biochemistry, J N Medical College and Hospital, Aligarh Muslim University, Aligarh, 202002 India; 5 Center of Ocular Inflammation and Infection, Laura Bassi Centres of Expertise (OCUVAC), Institute of Specific Prophylaxis and Tropical Medicine [ISPTM], Center for Pathophysiology, Infectiology and Immunology [CePII], Medical University of Vienna, Kinderspitalgasse 15, A-1090 Vienna, Austria

**Keywords:** Naringenin, Potassium bromate, Nephrotoxicity, Stress, Rats

## Abstract

The usage of potassium bromate (PB) in various consumer items and food and chemical industries poses a serious health risk like nephrotoxicity along with other toxic abuses. The compound elicits mild to severe toxic insults in a dose-dependent way in vivo and can even trigger carcinogenesis if exposed for long. This investigation ascertains the protective effect of naringenin (NIR), a polyphenol, in the PB-challenged rats. The animals were categorized into five treatment groups: Group I (control), and Groups II and III were treated with PB alone (100 mg/kg) and NIR alone (20 mg/kg), respectively. The groups– IV and V were administered with NIR at 20 and 40 mg/kg in the PB-exposed rats. The animals were killed to get their kidney and blood samples for biochemical, molecular, and histological alaysis. The group II exhibited an enhanced level of renal function and toxicity markers in serum, validating nephrotoxicity. Further, dysregulated redox parameters (GSH and MDA) and suppressed antioxidant enzymes (SOD and CAT) consolidated the same. Group III showed negligible toxicity, with most of the parameters' values close to the control. Intriguingly, groups- IV and V bestowed remarkable alleviation in the PB-induced nephrotoxicity in a dose -dependent way. In addition, the comet assay and histological evaluation further confirmed these findings. The study concludes that NIR effectively protects against PB-induced nephrotoxicity in rats attributed by regulating oxidative stress and promoting structural restoration at both macromolecular and cellular levels. Therefore, NIR is a suitable candidate for use in consumer products containing PB or as a treatment to alleviate nephrotoxicity caused by PB or similar compounds.

## Introduction

Xenobiotics are compounds that are either not naturally found in the body or are present at higher concentrations than typical physiological levels. These include environmental toxins, pharmaceuticals, industrial chemicals, cosmetics, and dietary additives (Al-Tamimi et al. 2018; Lang et al. 2025). As modern society is exposed to these potentially harmful substances more frequently through food, drink, and the air, a new field of toxicology is emerging to study how xenobiotics affect human health. Risk assessment and the development of effective public health policies depend on understanding the harmful effects of xenobiotics (Hassan et al. 2013).

One of the controversial xenobiotics is potassium bromate (KBrO₃; PB), which has been widely used in various industries, notably in paints, colors, food and beverages, and cosmetics (Hassan et al. 2020a). Its primary purpose is to strengthen and increase the flexibility of dough, helping baked goods rise and improve their texture. The International Agency for Research on Cancer and the Environmental Protection Agency (EPA) have classified it as a possible carcinogen (Environmental Protection Agency (EPA) 2021). Its metabolic waste products can harm the kidneys and liver, among other major health concerns. After consumption and digestion, bromate ions are produced through metabolism, leading to oxidative stress in vivo. This oxidative stress has been linked to cellular damage, inflammation, and the initiation of carcinogenesis (Al-Mareed et al. 2022). Indicators of nephrotoxic damage associated with increased exposure to potassium bromate include elevated liver enzyme levels, histological changes, and neoplastic transformations (Schoeny and McCormick 2020). Although potassium bromate is known to be harmful, it remains permitted and is commonly used in the United States and other countries. However, several other nations have banned it. Regulatory agencies such as the Environmental Protection Agency (EPA) and the U.S. Food and Drug Administration (FDA) recognize the potential risks of potassium bromate; nonetheless, enforcement and compliance vary significantly across jurisdictions (Food and Drug Administration 2021). This regulatory discrepancy underscores the need to reevaluate its safety, especially considering possible public health implications. The compound has been reported to elicit major toxic insults, including nephrotoxicity while several natural compounds have been reported to counter nephrotoxicity (Hassan et al. 2020b; Bayomy et al. 2016).

Herbal extracts are increasingly regarded as a powerful natural alternative to synthetic pharmaceuticals (commercial medicines), primarily due to their dense concentrations of antioxidant and anti-inflammatory bioactive compounds (Bayomy et al. 2016; Ahmed et al. 2025). By targeting oxidative stress and chronic inflammation—the fundamental biological drivers underlying most diseases and allergic reactions—these extracts offer a holistic therapeutic approach. They effectively regulate redox homeostasis and inflammatory responses, thereby strengthening the immune system and accelerating the healing process across a wide range of clinical conditions (Ahmed et al. 2025). These herbs derive therapeutical properties from their bioactive components like flavonoids, polyphenols, terpenoids, and alkaloids Among such active compounds, naringenin, a naturally existing citrus fruit flavonoid, is dependent upon a three-carbon bridge connecting its two aromatic rings (Parham et al. 2020). This interesting flavonoid has been implicated in several health perks, especially in connection with its anti-inflammatory, antioxidant, and metabolic regulatory properties. Because of its multiple physiological functions and its health benefits, naringenin has been probed by the pharmaceutical and nutritional sciences (Ahmed et al. 2025). This compound originated from the aromatic amino acid phenylalanine and is typically conjugated as glycosylated, neohesperidoside, and aglycone (Kumar and Pandey 2013). Additionally, this chemical has been linked to anti-inflammatory and cholesterol-lowering effects and enhances blood vessel endothelial function (Wang et al. 2018). The chemical's antioxidant and anti-inflammatory capacities have been additionally linked to neurodegenerative disease prevention (Adetunji et al. 2023). The flavonoid most prominently exhibits anti-cancer properties via modifying many signalling pathways that promote tumor formation. Studies demonstrate that it may inhibit cancer cell lines from multiplying, which triggers apoptosis (Gao et al. 2019). Despite its considerable first-pass metabolism, the molecule's poor bioavailability makes it challenging to reap all of its therapeutic benefits (Choi et al. 2018; Bae et al. 2020). Researchers are currently examining the compound's efficacy against a range of hazardous substances that affect the environment and biology. The current study seeks to ascertain if NIR can ameliorate the nephrotoxicity caused by PB in vivo.

## Materials and methods

### Materials

Most of the chemicals and reagents were procured from Millipore Merck (Darmstadt, Germany), Biolab (UK), and Sigma Aldrich (St. Louis, Missouri, USA). We procured most of the biochemical assay and estimation kits from Quimica Clínica Aplicada (Spain).

### Methods

#### Experimental animal treatment strategies

Thirty male healthy Swiss albino rats (3 months old) were acquired from the Central animal house (Department of Zoology), KSU, Riyadh. These rats were housed in commercial plastic cages in typical controlled conditions, which included a 12-h day/night cycle, a relative humidity of 72–77%, and a temperature of 22 ± 3 °C. They received regular rat meal pellets and unrestricted access to clean tap water.

Five groups of animals have been chosen at random from among them:

- Group 1: vehicle control treated with saline.
- Group 2: One dose of potassium bromate [CAS no. 7758–01–2 (Sigma-Aldrich, US), purity 99.8%] at an administration rate of 100 mg/kg body weight (Hassan et al. 2020a; Bayomy et al. 2016).
- Group 3: naringenin [CAS no. 67604–48-2 (Sigma-Aldrich, US), purity 98%] thrice a week for a month at dosage of 20 mg/kg body weight (Sahu et al. 2019).
- Group 4: One dose of potassium bromate at 100 mg/kg body weight, followed by two weekly doses of naringenin at 20 mg/kg body weight for 30 days,
- Group 5: A single dosage of potassium bromate at 100 mg/kg body weight followed by naringenin at 40 mg/kg body weight twice each week for 30 days.

The current investigation employed intraperitoneal delivery for all test drugs. Samples were acquired by sacrificing the animals on the same day the therapy concluded as described by IACUC (Institutional Animal Care and Use Committee, University of Iowa, 2023). The study approved under KSU-SE-20–69 has been reviewed by the Institutional Ethical Committee.

#### Sample designing

The kidney samples were rinsed with cooled phosphate-buffered saline (PBS) and then centrifuged in Tris-KCl buffer (pH 7.36; Ika-Werke, Germany) to get the supernatant (Gao et al. 2019). The sera were collected by centrifugation at 1000 × g (Eppendorf, Germany) and then stored in a refrigerator. Histopathological and comet assay investigations have been conducted on half of the kidney samples.

#### Assessing renal functionality

Albumin (Cat.No. 997258), urea (Cat. No. 991305), creatinine (Cat. No. 998891), and BUN (Cat. No. 991305) were assessed as per the instructions provided by the commercially available assay kits (Química Clínica Aplicada diagnostic kits, Spain).

#### Assessing renal toxicity

Glutathione S-transferase (GST, Cat. No. 991056) was assessed as per the instructions provided by the commercially available assay kit, (Quimica Clinica Aplicada S.A., Spain).

#### Assessing oxidative stress scenario

Critical antioxidant enzymes, such as superoxide dismutase (SOD) and catalase (CAT), were assessed as per the instructions mentioned in the well-known standard methods (Aebi 1984; Marklund and Marklund 1974).

#### Assessing redox status

Reduced Glutathione (GSH) level and total malondialdehyde (MDA) were assessed as per the instructions mentioned in the well-known standard methods of Jollow et al. (Jollow et al. 1974) and Beuge and Aust (Buege and Aust 1978).

#### Assessing lipid profile patterns

Serum cholesterol (Cat. No.995280), TAGs (Cat. No. 992320), HDL (Cat. No. 998058), and LDL (Cat. No. 991880) were assessed as per the instructions provided by the commercially available assay kits (Química Clínica Aplicada kits, Spain).

#### Assessing nitrosative stress

The commercially available QuantiChrom TM Bioassay system Nitric Oxide Assay kit, (Cat. No. DINO-250, Hayward, CA94545, USA), that employs the Griess reagent at 540 nm, has been utilized for evaluating nitric oxide (NO) in accordance with the recommendations.

#### Assessing necrosis as a cell death marker

Lactate dehydrogenase (LDH, Cat. No. 990035) activity was assessed as per the instructions provided by the commercially available assay kits (Quimica Clinica Aplicada, S.A., Spain) to evaluate the degree of necrosis (Chan et al. 2013).

#### Histological analysis

The kidney tissue samples were stored in an 8% formaldehyde solution and processed and snapped as previously done (Hassan et al. 2025).

#### Comet assay

This assay of the kidney cell suspension was conducted in alkaline conditions using the standard procedure as done before (Hassan et al. 2012).

##### Statistical analysis

All the data has been analyzed and expressed as mean ± SD by using GraphPad Prism 5 software. After a one-way ANOVA analysis of the data, Tukey's post hoc multiple comparison test was implemented. P values below 0.05 were considered statistically significant in this present study. The marks “*, **, and ***” were used as asterisks to indicate significant differences from the negative control (CN −, group I) at *p* ≤ 0.05, 0.005, and 0.001, while the marks “#, ##, and ###” indicated significant differences from the positive control (CN +, group II) at the same *p*-values.

## Results

### Effect on renal function markers

#### Albumin

**Fig. 1:**
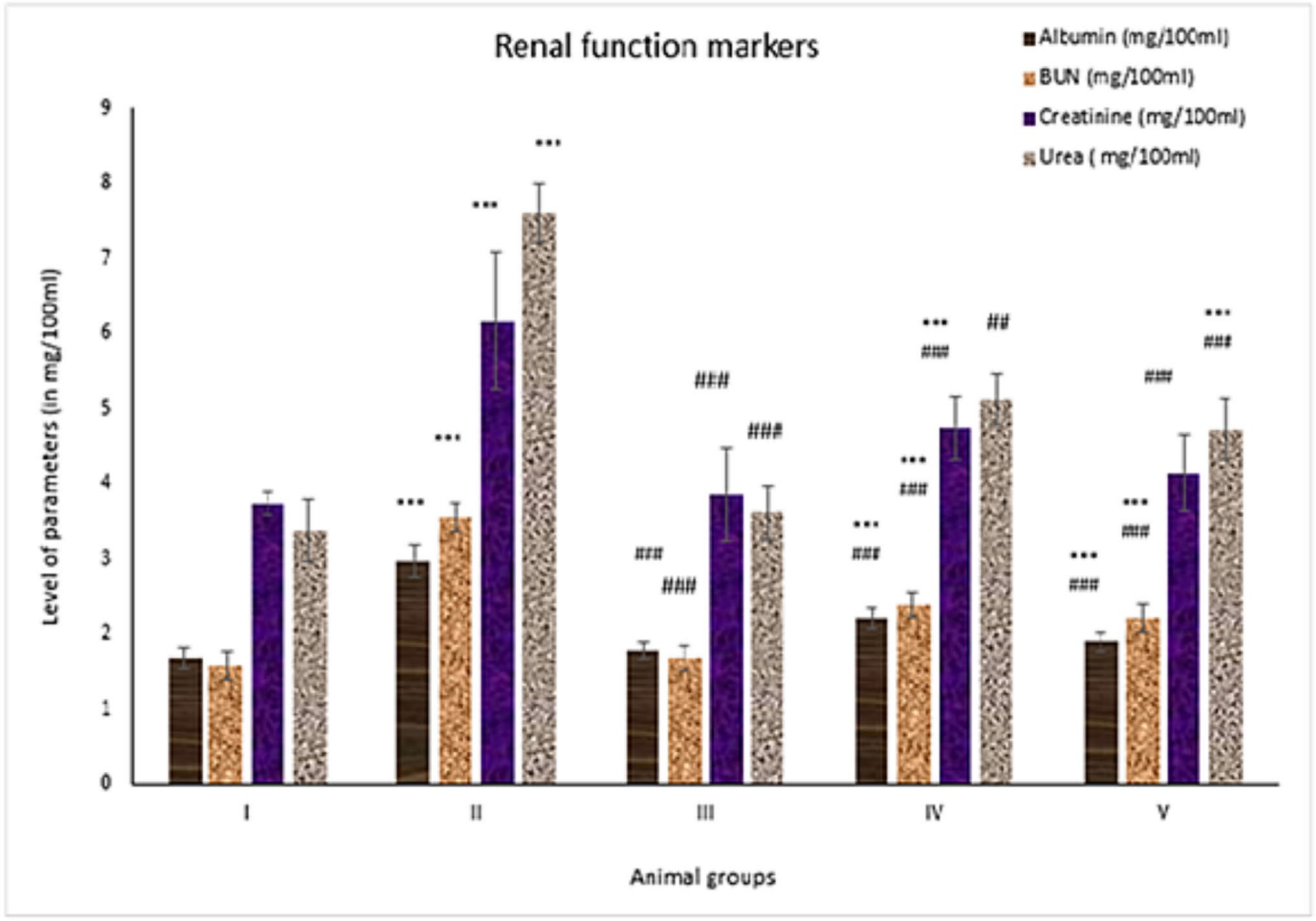
Bar graph showing the renal function markers (albumen, blood urea nitrogen, creatinine and urea) in serum samples from the treatment groups- I (Control), II (Potassium bromate, PB, treated), III (Naringenin, NIR, treated); IV (PB + NIR 1) and V (PB + NIR2). All the data have been expressed as the mean ± SD of six independent experiments. The marks “***” were used as asterisks to indicate significant differences from the negative control (CN −, group I) at *p* ≤ 0.001, while the marks “###” indicated significant differences from the positive control (CN +, group II) at the same *p*-values

Group II showed an elevation in its level by 77.52% compared to the control (*p*-value ≤ 0.001), Group I, while Group III showed an increase of 6.87%. However, Groups IV and V demonstrated a decline in its level by 25.22% and 36.06% (*p*-value ≤ 0.001) compared to Group II (Fig. 1).

#### Urea

It is one of the primary renal markers for assessing kidney function in vivo. Groups II and III demonstrated an increase in their levels by 125.26% (p-value ≤ 0.001) and 7.25%, respectively, as compared to the control, Group I (control). However, Groups IV and V exhibited declined levels by 32.62% and 37.84% (p-value ≤ 0.001) compared to the positive control, Group II (Fig. 1).

#### Creatinine

Another significant renal measure for evaluating renal function is creatinine. Group II displayed a rise in its level by 64.86% (*p*-value ≤ 0.001) after treatment with PB with respect to the control, Group I, while Group III exhibited enhanced levels by 3.04% compared to the control. Hitherto, Groups IV and V showed a decline in their level by 23.14% (*p*-value ≤ 0.01) and 32.86% (*p*-value ≤ 0.001) (Fig. 1).

#### BUN

The mice from Groups II and III showed an increase in level by 125.30% (p-value ≤ 0.001) and 7.29% with respect to Group I, while Groups IV and V exhibited decreased levels by 32.64% and 37.85% (*p*-value ≤ 0.001) as compared to the positive control, Group II (Fig. 1).

### Effect on renal toxicity markers

#### Glutathione S-transferase (GST)

**Fig. 2:**
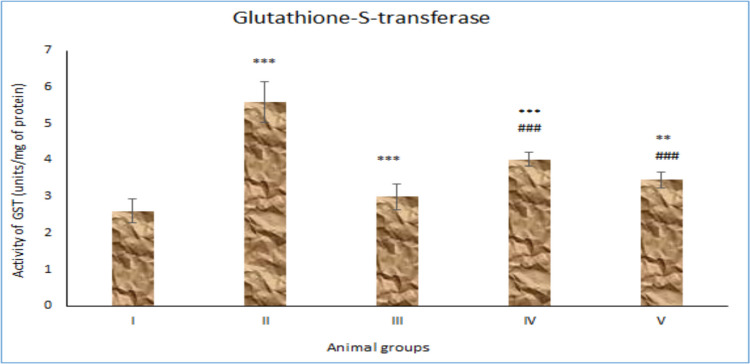
Bar graph showing the activity of GST of different treatment groups- I (Control), II (Potassium bromate, PB, treated), III (Naringenin, NIR, treated); IV (PB + NIR 1) and V (PB + NIR2). All the data have been expressed as the mean ± SD of six independent experiments. The marks “***” were used as asterisks to indicate significant differences from the negative control (CN −, group I) at *p* ≤ 0.001, while the marks “###” indicated significant differences from the positive control (CN +, group II) at the same *p*-values

Groups II and III displayed an increase in its level by 115.0% (p-value ≤ 0.001) and 15.34% with respect to the control (Group I), while Groups IV and V demonstrated a decrease in its level by 28.21% (p-value ≤ 0.001) and 38.16% (p-value ≤ 0.01) with respect to Group II (Fig. 2).

### Effect on antioxidative enzymes

#### Superoxide dismutase (SOD)

**Fig. 3:**
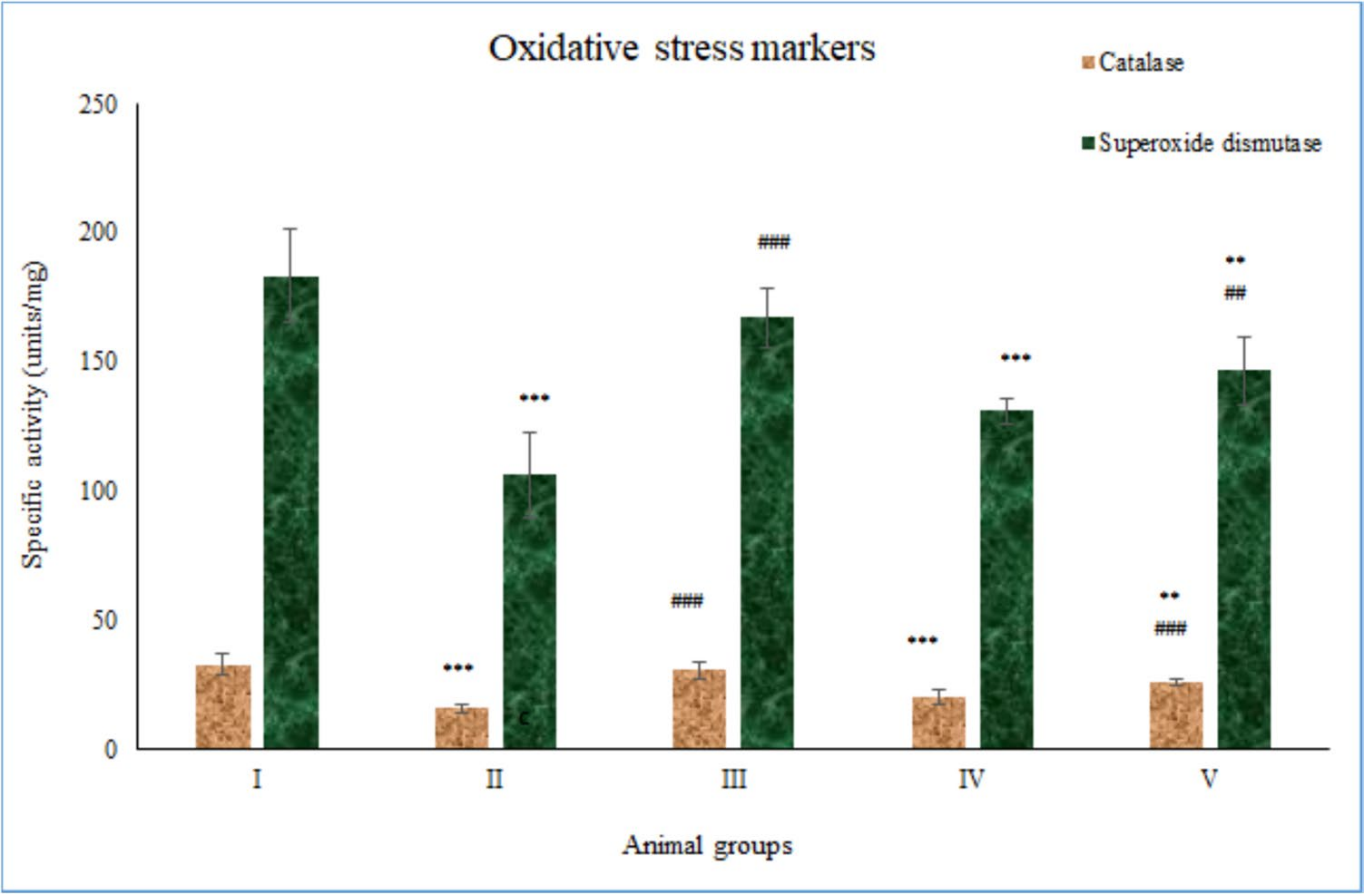
Bar graph showing the activity of oxidative stress markers (SOD and CAT) of different treatment groups- I (Control), II (Potassium bromate, PB, treated), III (Naringenin, NIR, treated); IV (PB + NIR 1) and V (PB + NIR2). All the data have been expressed as the mean ± SD of six independent experiments. The marks “, **, and ***” were used as asterisks to indicate significant differences from the negative control (CN −, group I) at *p* ≤ 0.005, and 0.001, while the marks “, ##, and ###” indicated significant differences from the positive control (CN +, group II) at the same *p*-values

Group II and III after PB treatment showed depletion in its activity by 42.03% (p-value ≤ 0.001) and 8.88% compared to the control, Group I. However, the combination-treated Groups IV and V had increased activity of it by 23.33% (p-value ≤ 0.001) and 37.9% (*p*-value ≤ 0.01), compared to Group II (Fig. 3).

#### Catalase (CAT)

Figure shows the activity of CAT. Groups II and III showed a compromised level of CAT by 51.56% (*p*-value ≤ 0.001) and 7.26% in comparison to the control. However, the combination of Groups IV and V showed enhancement in its activity by 26.59% and 61.85% (p-value ≤ 0.001) compared to Group II (Fig. 3).

### Effect on Redox parameters

#### Reduced Glutathione (GSH)

**Fig. 4:**
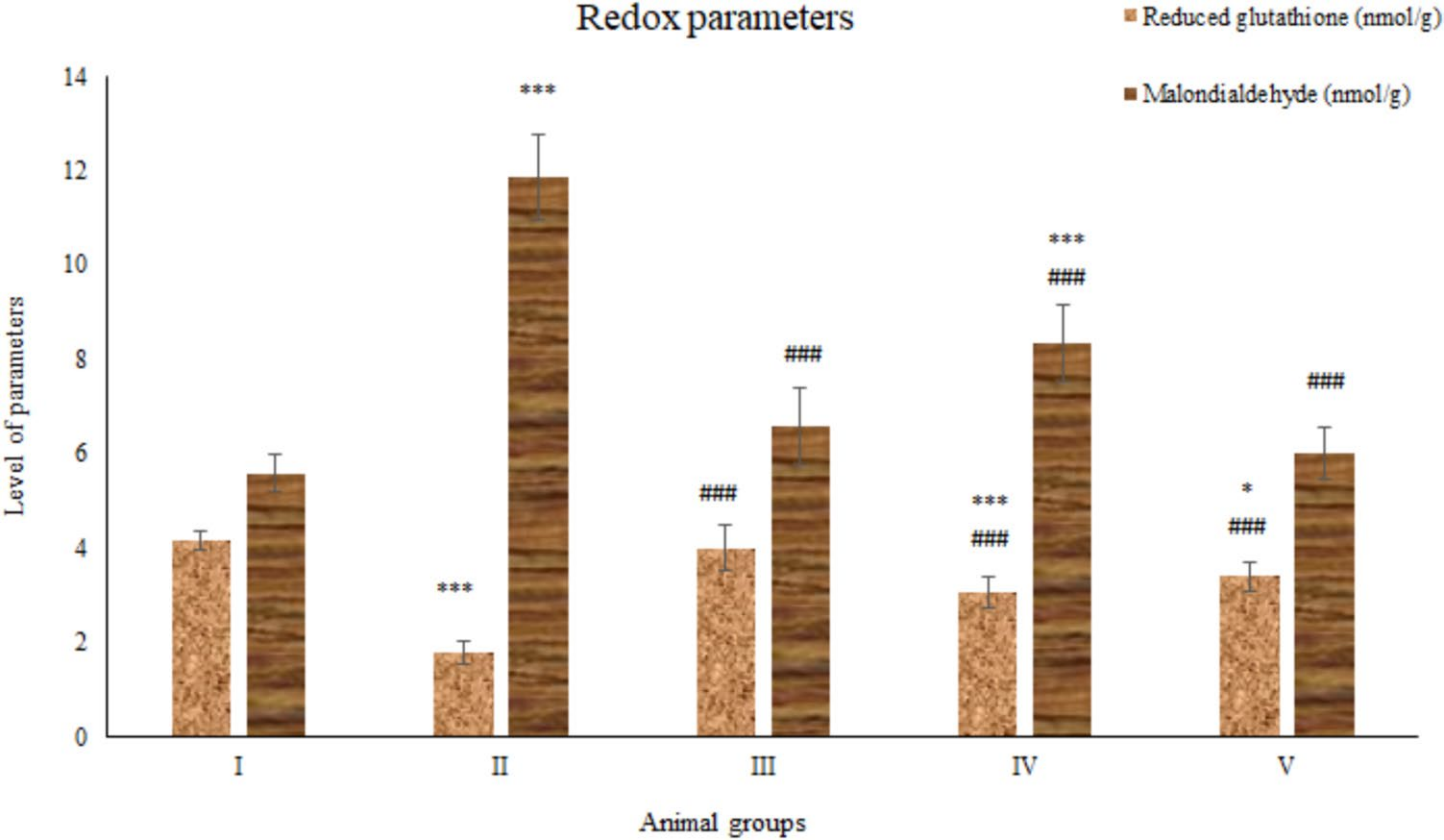
Bar graph showing the activity of redox parameters (GSH and MDA) of different treatment groups- I (Control), II (Potassium bromate, PB, treated), III (Naringenin, NIR, treated); IV (PB + NIR 1) and V (PB + NIR2). All the data have been expressed as the mean ± SD of six independent experiments. The marks “*, **, and ***” were used as asterisks to indicate significant differences from the negative control (CN −, group I) at p ≤ 0.05, 0.005, and 0.001, while the marks “#, ##, and ###” indicated significant differences from the positive control (CN +, group II) at the same *p*-values

A decline of 56.77% (*p*-value ≤ 0.001) and 3.78% was observed in Groups II and III, respectively, in the GSH level in comparison to the control. However, the treatment with the combinations of PB with naringenin caused its replenishment by 69.9% (*p*-value ≤ 0.001) and 89.42% (*p*-value ≤ 0.001) compared to Group II (Fig. 4).

#### Malondialdehyde (MDA)

One of the most trustworthy biochemical indicators for determining lipid peroxidation is MDA. The level of MDA was found to have increased by 112.16% (*p*-value ≤ 0.001) in Group II, followed by 7.29% in Group III. However, Groups IV and V showed a decline in their levels of 29.51% (*p*-value ≤ 0.001) and 49.32% (*p*-value ≤ 0.001) compared to Group II (Fig. 4).

### Effect on lipid profile parameters

#### Serum cholesterol

**Fig. 5:**
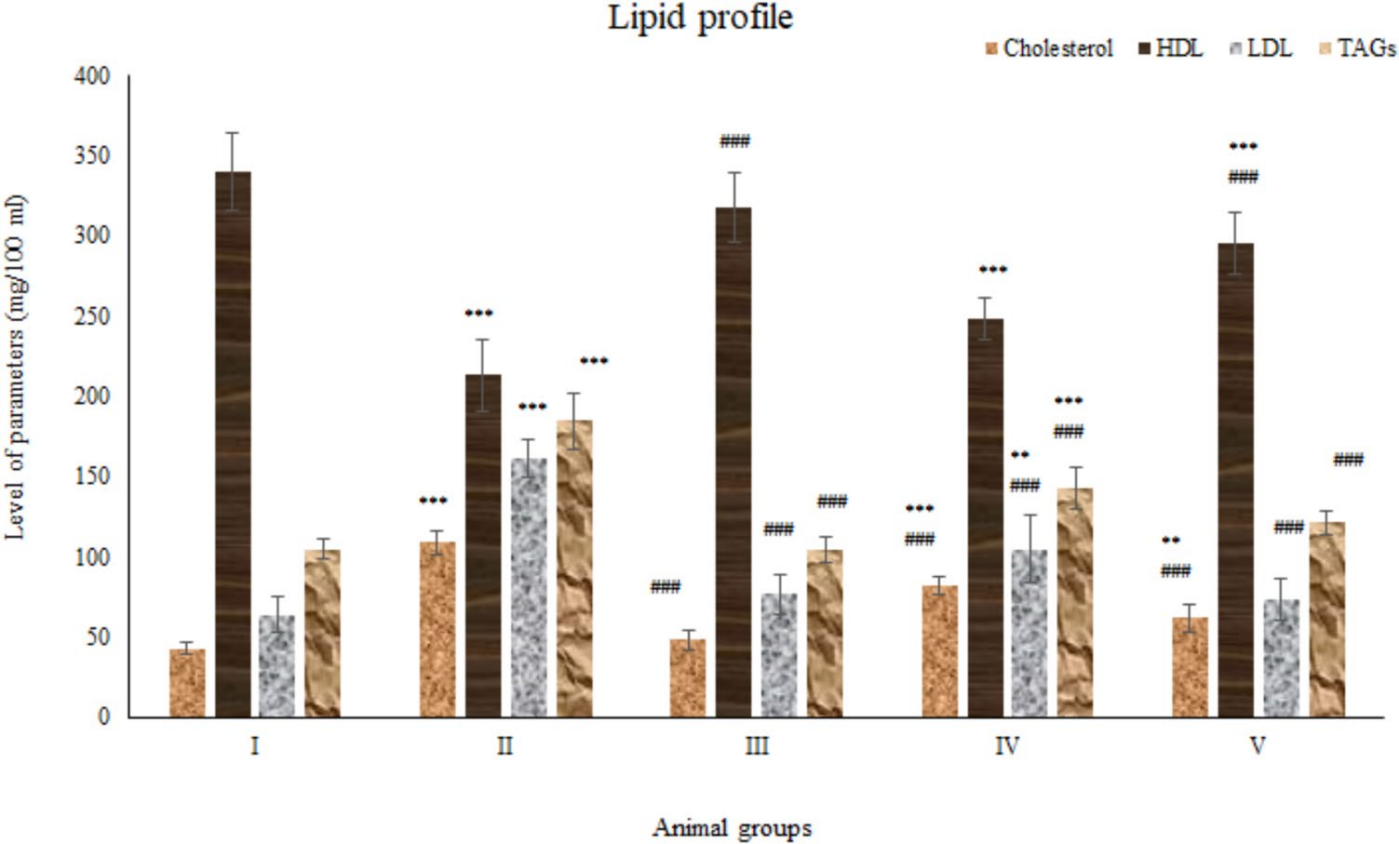
Bar graph showing the activity of lipid profile (Cholesterol, TAGs, LDL and HDL) of different treatment groups- I (Control), II (Potassium bromate, PB, treated), III (Naringenin, NIR, treated); IV (PB + NIR 1) and V (PB + NIR2). All the data have been expressed as the mean ± SD of six independent experiments. The marks “*, **, and ***” were used as asterisks to indicate significant differences from the negative control (CN −, group I) at *p* ≤ 0.05, 0.005, and 0.001, while the marks “#, ##, and ###” indicated significant differences from the positive control (CN +, group II) at the same *p*-values

Groups II, and III displayed an increase in its level by 153.66% (*p*-value ≤ 0.001), and 12.33% with respect to the control (Group I), while Groups IV and V demonstrated a decrease in its level by 24.61% (*p*-value ≤ 0.001) and 42.74% (*p*-value ≤ 0.001) with respect to Group II (Fig. 5).

#### TAGs

Group II showed an increase in its level by 75.71% (*p*-value ≤ 0.001) while group III, showed decline in its activity by 0.48% compared to group I. However, group IV and V showed decline in its activity by 22.44% (*p*-value ≤ 0.001) and 34.2% (*p*-value ≤ 0.001) respectively (Fig. 5).

#### HDL

The animal Groups II, and III, demonstrated dip in its level by 37.27% (*p*-value ≤ 0.001), and 6.48% after treatment with PB, with respect to Group I, while the combination-treated Groups IV and V exhibited an increase in its level by 16.26% (*p*-value ≤ 0.01) and 38.38% (p-value ≤ 0.001) as compared to Group II (Fig. 5).

#### LDL

Groups II showed an increase by 152.45% (*p*-value ≤ 0.001), whereas group III showed decrease 20.0% after treatment with PB, with respect to Group I. Furthermore, Groups IV and V demonstrated a decrease in its level by 35.13% (*p*-value ≤ 0.001) and 54.35% (*p*-value ≤ 0.001) as compared to Group II (Fig. 5).

### Effect on nitrosative stress parameters

#### NO

**Fig. 6:**
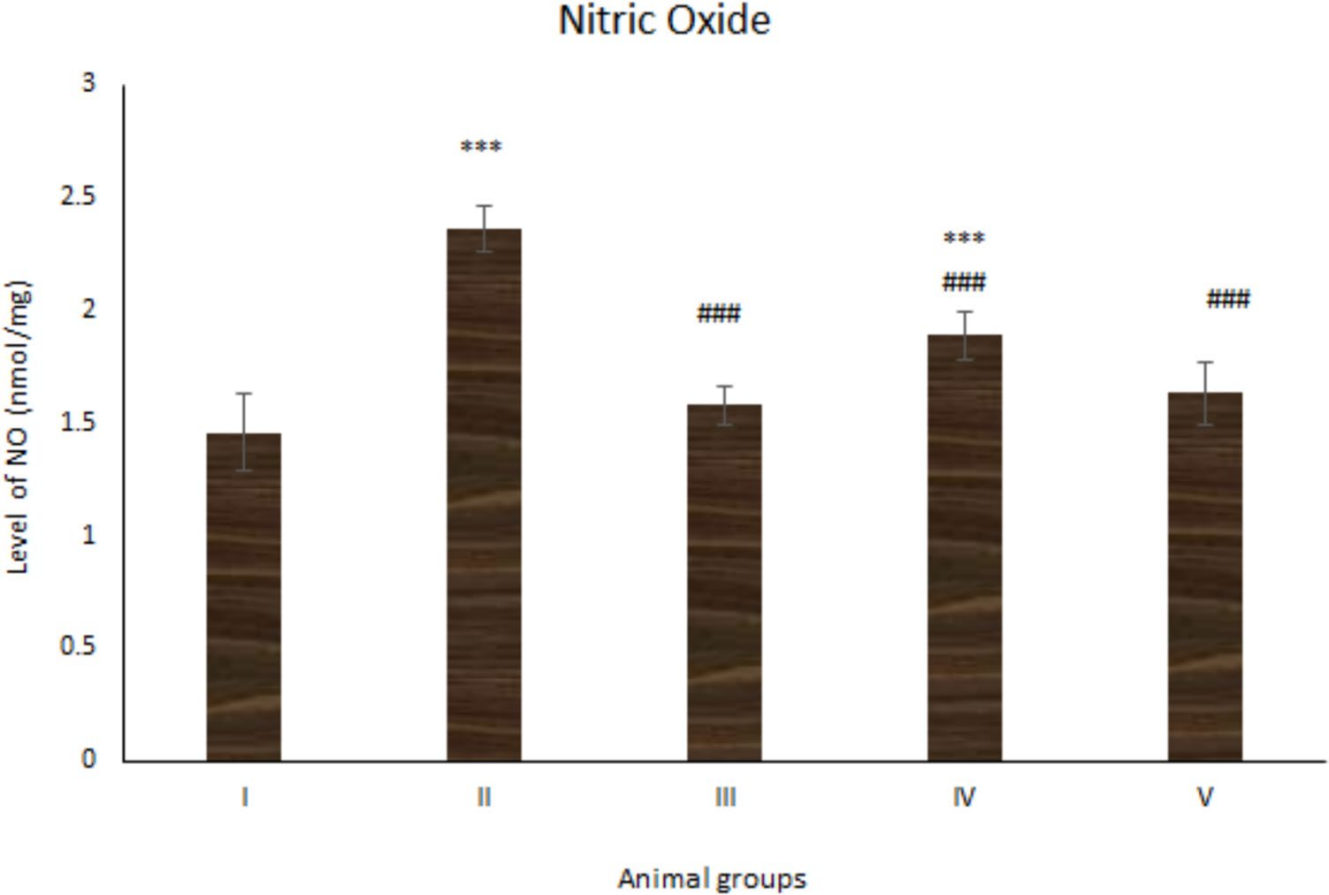
Bar graph showing the level of NO of different treatment groups- I (Control), II (Potassium bromate, PB, treated), III (Naringenin, NIR, treated); IV (PB + NIR 1) and V (PB + NIR2). All the data have been expressed as the mean ± SD of six independent experiments. The marks “*, **, and ***” were used as asterisks to indicate significant differences from the negative control (CN −, group I) at *p* ≤ 0.05, 0.005, and 0.001, while the marks “#, ##, and ###” indicated significant differences from the positive control (CN +, group II) at the same *p*-values

Groups II, and III displayed an increase in its level by 62.16% (*p*-value ≤ 0.001), and 8.43% with respect to the control (Group I), while Groups IV and V demonstrated a decrease in its level by 20.11% (*p*-value ≤ 0.001) and 30.89% (*p*-value ≤ 0.001) with respect to Group II (Fig. 6).

### Effect on comet assay

The treatment showed significant effect on nuclear DNA of the kidney cells from various groups. PB- treated Group II demonstrated increase in tail-length by 70.89% (*p*-value ≤ 0.001) as compared to the control, group I while group III displayed negligible nuclear damage quite comparable to the control. However, group IV and V exhibited dose-dependent decrease by 20.92% (*p*-value ≤ 0.001) and 35.40% (*p*-value ≤ 0.001) respectively in comparison to group II. respectively (Table 1).

**Table 1:**
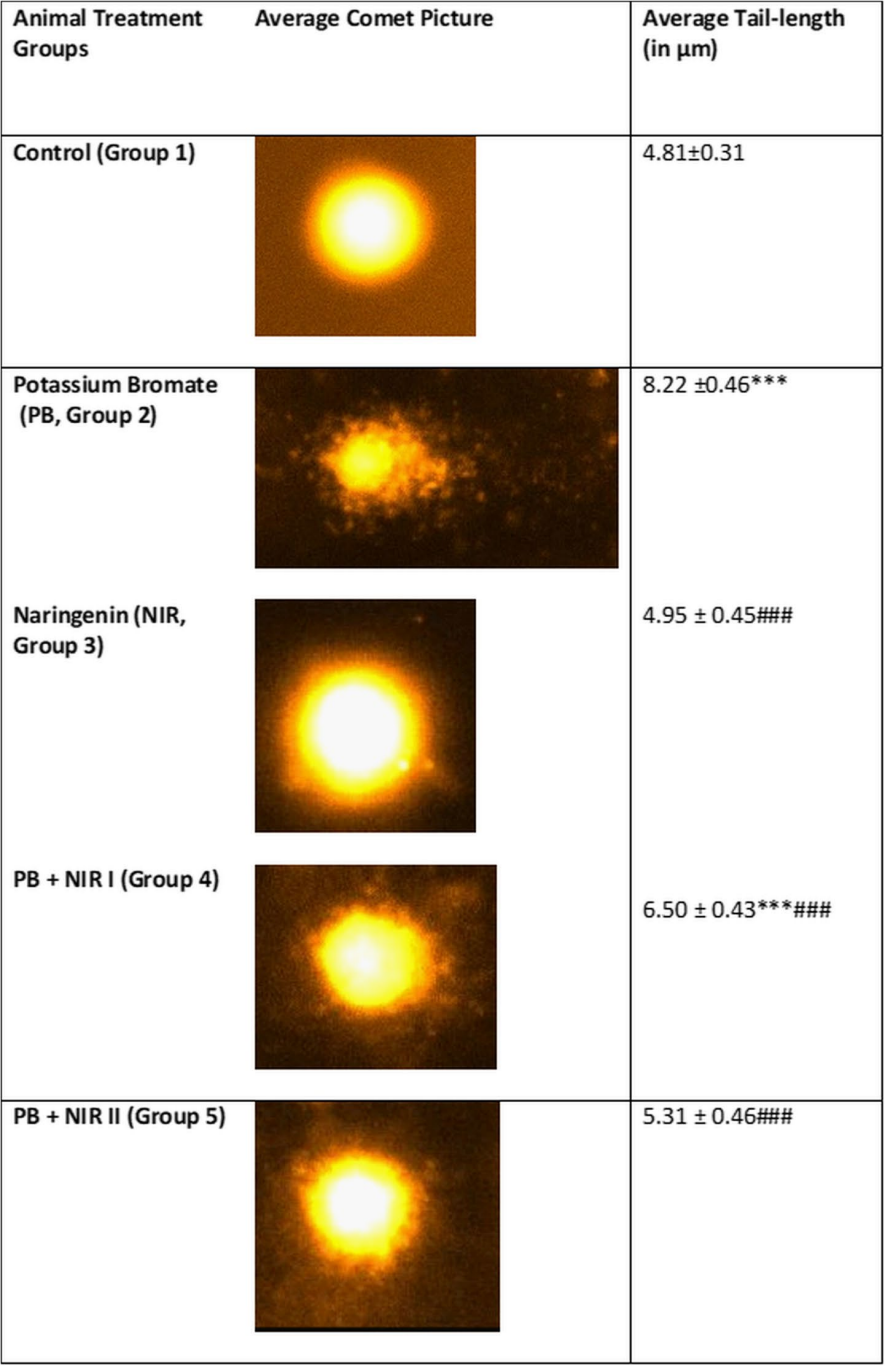
Showing the average comet picture of kidney cells from the treated groups along with comet tail-length measured in µm by Comet 5.5 software (Andor, Oxford)

The marks “*, **, and ***” were used as asterisks to indicate significant differences from the negative control (CN −, group I) at *p* ≤ 0.05, 0.005, and 0.001, while the marks “#, ##, and ###” indicated significant differences from the positive control (CN +, group II) at the same *p*-values

### Histopathological and morphometric analysis

**Fig. 7:**
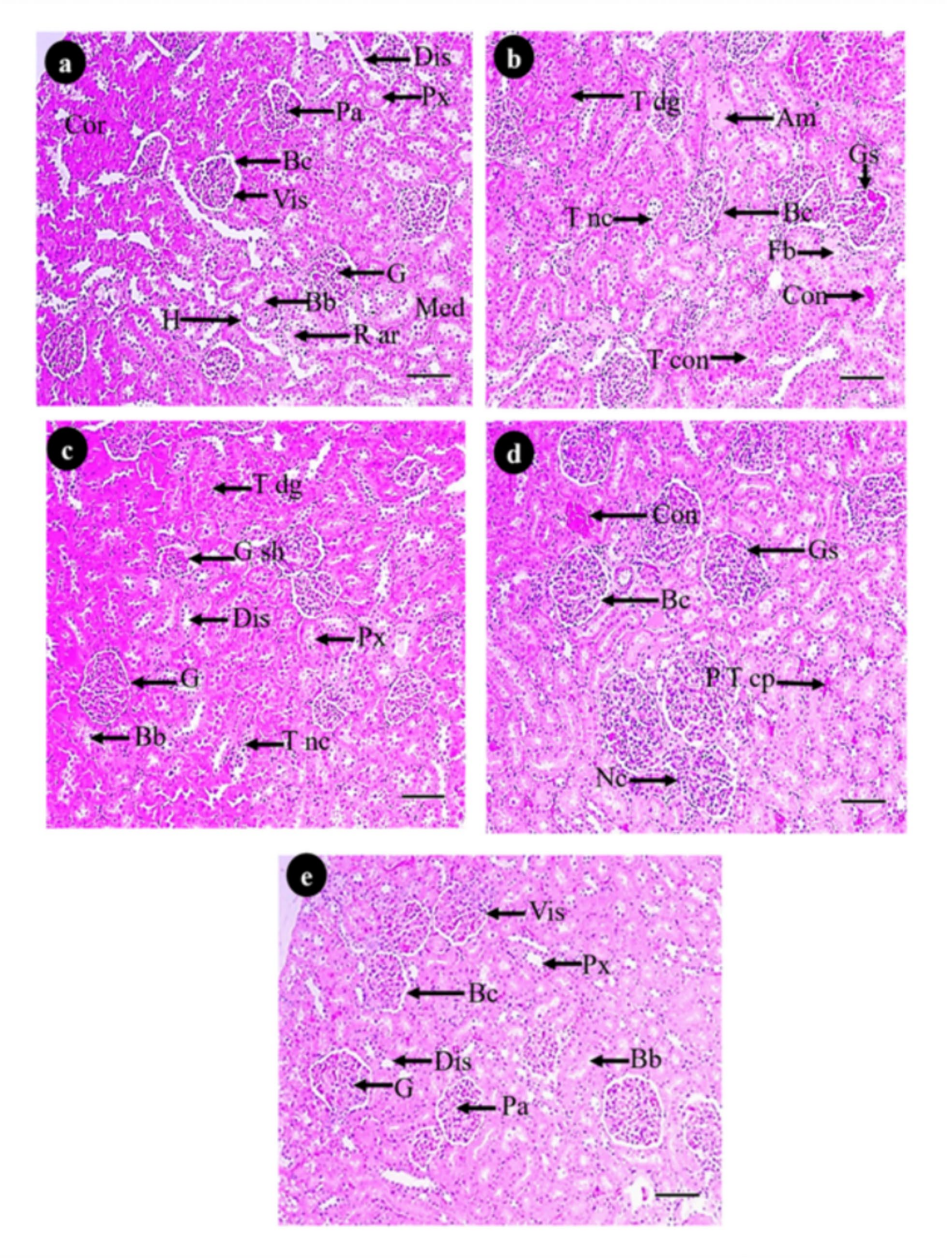
Photomicrograph of the histological section (H&E staining) of different treatment groups- I (Control, 7a), II (Potassium bromate, PB, treated, 7b), III (Naringenin, NIR, treated, 7c); IV (PB + NIR 1, 7d) and V (PB + NIR2, 7e) showing the cortical area (Cor), a bowman’s capsule (Bc), glomerulus (G), visceral layer (Vs) and the partial layer (Pa), proximal convoluted tubules (Px) with brushing borders (Bb), distal convoluted tubules (Dis), renal artery (R ar), Henle loop (H), and medullary region (Med), tubular degeneration (T dg), amyloidlysis (Am), glomerulus sclerosis (Gs), fibrosis (Fb), congestion (Con), tubular necrosis (T nc), tubular congestion (T con), glomerular shrinkage (G sh), necrosis (Nc), peritubular capillaries (P T cp). All the pictures were snapped at 200 × by a digital camera (Nikon Dxm1200C model, Japan) with a resolution of 12.6 megapixels (4116 × 3072 pixels) attached to the microscope (Nikon Eclipse 80i, Japan) with image analyzing software Nikon’s ACT-1C. The line ‘ —’ shown in pictures measures 200 µm

**Fig. 8:**
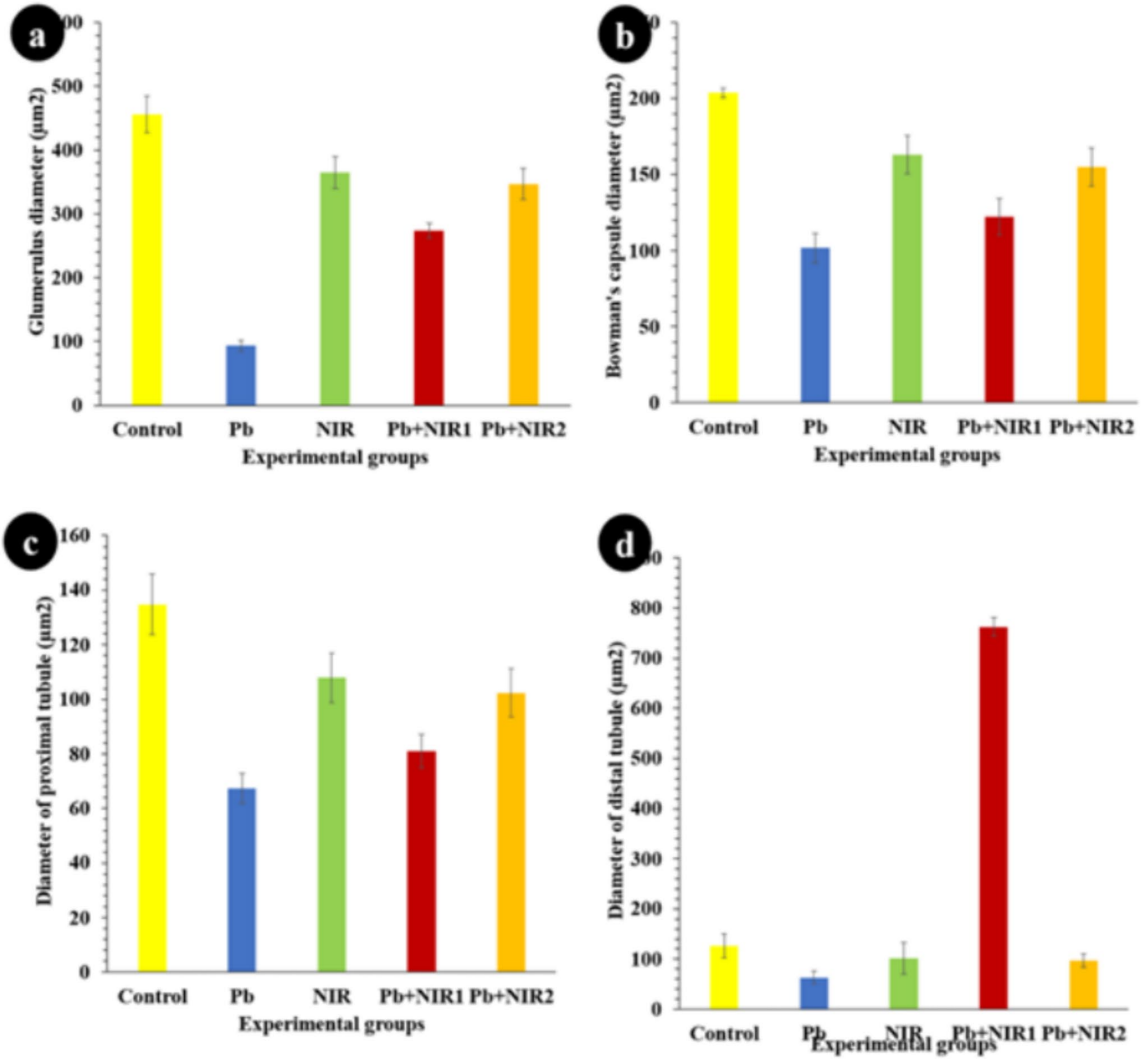
Bar charts showing the morphometric analysis of renal tissue. The histomorphometric parameters are (**a**) diameter of glomerulus, (**b**) Bowman’s capsule diameter, (**c**) diameter of proximal tubule, (**d**) diameter of distal tubule, among five experimental groups: control, potassium bromates, naringenin, potassium bromates + naringenin 1, and potassium bromate + naringenin 2. Values are represented as Mean ± STDV & n = 10 animals. Means within the same parameter and not sharing a common superscript symbol(s) differ significantly at *p* < 0.05, and values that are recorded with a non-significant difference (n.s)

The histopathological examination combined with quantitative morphometric analysis revealed profound structural alterations in renal tissue across different experimental groups (Figs. 7, 8 and Table 2). The control kidneys demonstrated optimal renal architecture with large, well-formed glomeruli measuring 456.47 ± 108.66 μm^2^ in diameter, enclosed within intact Bowman's capsules of 203.67 ± 53.26 μm^2^. The cortical area showed healthy proximal convoluted tubules with prominent brush borders averaging 134.83 ± 55.17 μm^2^ in diameter, and well-defined distal convoluted tubules measuring 126.67 ± 23.83 μm^2^. The tissue exhibited minimal pathological changes with negligible global sclerosis (0.13 ± 0.01%), amyloid deposition (0.28 ± 0.034%), congestion (1.51 ± 0.07%), fibrosis (1.07 ± 0.063%), and tubular necrosis (1.13 ± 0.07%).

**Table 2:**
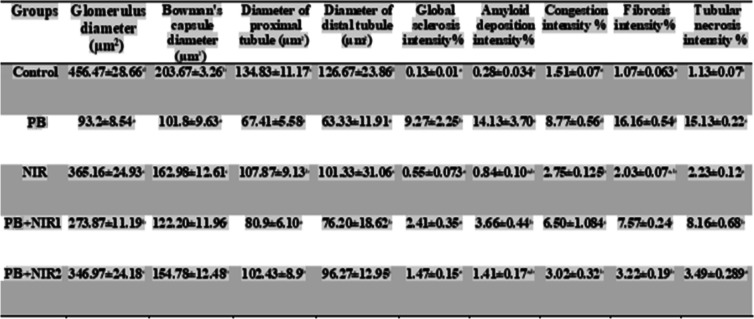
Showing the morphometric analysis of renal tissue

The histomorphometric parameters are diameter of glomerulus, Bowman’s capsule diameter, diameter of proximal tubule, diameter of distal tubule, glomerulus sclerosis intensity, amyloid deposition intensity, congestion intensity, fibrosis intensity, tubular necrosis intensity, among five experimental groups: control, potassium bromates, naringenin, potassium bromates + naringenin 1, and potassium bromate + naringenin 2. Values are represented as Mean ± STDV & *n* = 10 animals. Means within the same parameter and not sharing a common superscript symbol(s) differ significantly at p < 0.05, and values that are recorded with a non-significant difference (n.s)

Potassium Bromate renal tissue exhibited severe nephrotoxic alterations characterized by dramatically shrunken glomeruli reduced to 93.2 ± 21.54 μm^2^ (79% reduction from controls) and compressed Bowman's capsules measuring only 101.8 ± 19.63 μm^2^ (~ 50% reduction). The histological sections revealed extensive tubular degeneration, pronounced amyloidosis, glomerular sclerosis, widespread fibrosis, vascular congestion, tubular necrosis, and tubular congestion. Morphometric analysis confirmed severe structural damage with proximal tubule diameter reduced to 67.41 ± 12.58 μm^2^ and distal tubules to 63.33 ± 11.91 μm^2^ (approximately 50% reduction for both). Pathological intensity scores reached critical levels: global sclerosis (9.27 ± 2.25%), amyloid deposition (14.13 ± 3.70%), congestion (8.77 ± 1.56%), fibrosis (16.16 ± 3.54%), and tubular necrosis (15.13 ± 3.22%).

Naringenin administration alone showed remarkable preservation of renal architecture with minimal pathological changes. Glomerular diameter (365.16 ± 84.93 μm^2^) remained close to control values, representing only a 20% reduction compared to the 79% reduction seen with potassium bromate alone. Bowman's capsule diameter (162.98 ± 52.61 μm^2^) and tubular dimensions (proximal: 107.87 ± 44.13 μm^2^, distal: 101.33 ± 31.06 μm^2^) demonstrated good preservation. The tissue showed mild tubular degeneration and slight glomerular shrinkage, but pathological intensity scores remained low and comparable to controls.

Potassium Bromate + Naringenin 1 treatment demonstrated moderate therapeutic improvement with glomerular diameter recovering to 273.87 ± 51.19 μm^2^ (41% larger than PB group) and Bowman's capsule diameter increasing to 122.20 ± 41.96 μm^2^. However, morphometric analysis revealed an unusual finding in distal tubule measurements (762.20 ± 183.62 μm^2^), likely representing compensatory dilation. The histological sections showed reduced severity of glomerular shrinkage, decreased necrosis, and better preservation of peritubular capillaries Pathological scores showed partial improvement: global sclerosis (2.41 ± 0.35%), amyloid deposition (3.66 ± 0.44%), congestion (6.50 ± 1.084%), fibrosis (7.57 ± 1.24%), and tubular necrosis (8.16 ± 1.68%).

The higher naringenin dose provided optimal nephroprotection with near-complete restoration of renal architecture. Glomerular diameter recovered significantly to 346.97 ± 84.18 μm^2^ (76% of control values), while Bowman's capsule diameter reached 154.78 ± 72.48 μm^2^ (76% of controls). Tubular dimensions showed substantial recovery with proximal tubules measuring 102.43 ± 23.9 μm^2^ and distal tubules 96.27 ± 12.95 μm^2^. The histological appearance closely resembled control tissue with minimal visible pathological changes. Quantitative analysis confirmed near-normal pathological intensity scores: global sclerosis (1.47 ± 0.15%), amyloid deposition (1.41 ± 0.17%), congestion (3.02 ± 0.32%), fibrosis (3.22 ± 0.19%), and tubular necrosis (3.49 ± 0.289%) (Figs. 7, 8 and Table 2).

## Discussion

Multiple studies have demonstrated that naringenin has potent anticancer potential that can be employed to mitigate the adverse consequences of a variety of drugs and xenobiotics (Bilal et al. 2017). PB is present in several popular consumer products, including baked goods, packaged foods, cosmetics, and even drinking water. It is a documented health danger and a possible Group 2B carcinogen, according to the International Agency for Research on Cancer (IARC) (Ahmad and Mahmood 2012). The co-administration of natural substances and synthetic agents with therapeutic potential qualities has been recommended by numerous researchers as an approach to hinder detrimental effects of PB and enable its use with negligible/no health risks (Bilal et al. 2017; Ahmad and Mahmood 2012). PB produces reactive oxygen species via biotransformation that destabilize the cellular redox balance and structural integrity of target tissues (Yalçin and Çavuşoğlu 2022; Ahmad et al. 2015; Ali et al. 2018). Additionally, these radicals have the capacity to penetrate the membranes of the target cells and membrane-bound organelles such as the Golgi bodies, mitochondria, lysosomes, and endoplasmic reticulum. The biological activity of proteins, enzymes, lipids, and carbohydrates can be hampered by these substances, leading to malfunction and). Bromate and bromide are the highly disruptive metabolites that are produced via PB biotransformation and can result in harmful effects on macromolecules and cellular structures (Kawana et al. 1991; Alhazza et al. 2023). The primary objective of the current investigation was to ascertain whether naringenin treatment could mitigate these negative effects of PB in vivo. In the present study, naringenin demonstrated quite remarkable antioxidant capacity that is evident by a notable rise in antioxidant proteins and enzyme activity, along with a significant reduction in renal function parameters and associated toxicity indicators.

**Fig. 9:**
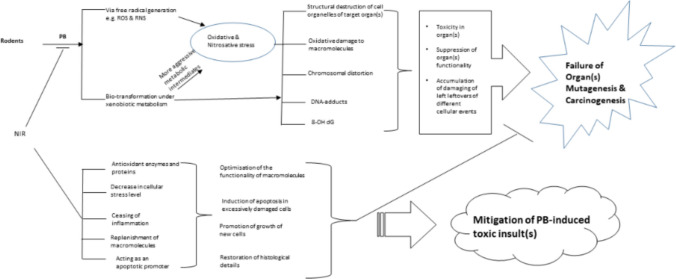
Showing the putative mechanism involving the major cellular and structural event during mitigation of potassium bromate (PB)-induced nephrotoxicity by naringenin (NIR) in vivo

The current work demonstrated the toxic influences in the experimental animals as evident by markedly increased renal function markers (albumin, urea, creatinine, and BUN) and a toxicity marker (GST). Nephrotoxicity results from the compound's accumulation in the kidneys and other important organs after prolonged exposure (Fig. 9). Serum samples from rats treated with PB revealed toxicity with renal indicators leaking because of the injury. Furthermore, the main culprit of an upsurge in oxidative damage to lipids and proteins (MDA) in the target organ was the free radicals generated by PB that further diminished the level of GSH and two other vital antioxidant enzymes, CAT and SOD. Numerous investigations based on animal and cell lines have proven that the toxicant PB elicits negative impacts in vivo that are accompanied by free radicals (Environmental Protection Agency (EPA) 2021; Al-Mosaibih 2025). Naringenin, on its counterpart's hand, serves as a natural antioxidant. Numerous investigations have revealed that the pure polyphenol is capable of more than just rendering multiple carcinogens inactive. The administration of naringenin to the rats that had previously received PB in the current research exhibited the reversed pattern. These findings correlate with other research findings that showed naringenin may enhance the activity of key antioxidant enzymes, such as CAT and GPx, along with SOD (Ali et al. 2018; Abdel-Latif et al. 2021). Hence, the biochemical evaluation including the renal function markers, toxicity marker and lipid profile clearly showed the significant trending towards the normal levels along with oxidative and nitrosative parameters in the PB-challenged rats treated with NIR in a dose-dependent way. Further, the comet assay also revealed that the nuclear DNA in the kidney cells from PB-treated group was highly damaged evidenced by long tail-length while NIR alone treated rat- kidney cells were similar to that of the control. However, PB-induced DNA damage was healed after NIR treatment in a dose dependent manner. All the results were also in agreement with the histological evaluation. The severe structural alterations observed in the potassium bromate group, including glomerular sclerosis, tubular necrosis, and interstitial fibrosis, are consistent with previous studies demonstrating bromate's ability to generate reactive oxygen species (ROS) and deplete antioxidant defenses (Hassan et al. 2020b; Valle-Velázquez et al. 2024). The significant reduction in glomerular and tubular diameters indicates cellular shrinkage and loss of functional nephrons, hallmarks of acute kidney injury progressing toward chronic kidney disease (Kawana et al. 1991; Huang et al. 2025).

NIR, a flavanone, is associated with remarkable protective properties through its potent antioxidant and anti-inflammatory activities (Hassan et al. 2025; Valle-Velázquez et al. 2024; Huang et al. 2025). The dose-dependent improvement observed in both histological architecture and quantitative parameters suggests that naringenin's protective effects involve multiple pathways such as antioxidant activity that represented the reduction in oxidative damage markers correlates with naringenin's ability to scavenge free radicals and enhance endogenous antioxidant enzyme activities (Wang et al. 2022; Alhazzani et al. 2025). On the other hand, the anti-inflammatory effects were evidenced by decreased congestion and reduced inflammatory cell infiltration suggesting naringenin's capacity to modulate inflammatory cascades and cytokine production (Fig. 9).

Several investigations have revealed the anti-fibrotic properties associated with naringenin's potential inhibiting fibroblast proliferation and collagen deposition, preventing progression to chronic kidney disease (Wang et al. 2022; Alhazzani et al. 2025). Also, it acted on the preservation of tubular brush borders and maintenance of glomerular architecture to suggest direct cytoprotective effects on renal cells (Alhazzani et al. 2025; Giradkar et al. 2024). The superior efficacy of the higher naringenin dose (NIR2) suggests a therapeutic window that could be optimized for clinical applications. The near-complete restoration of renal architecture and function parameters in the combination group higher dose of NIR indicates that it can serve as an effective therapeutic intervention for oxidative stress-mediated nephrotoxicity as recorded before (Peng et al. 2024) in rats.

The current investigation infers an immediate restrained regulatory intervention is required to control the public damage incurred by nephrotoxic effects of PB through various consumer items. The proposed flavanone, naringenin, is a perfect natural antidote to decrease the PB- induced nephrotoxic insults where PB usage is irreplaceable. In our previous study, we have shown that NIR can induce apoptosis in the entirely damaged cells and heal the mildly damaged cells by orchestrating the redox status and immunomodulation (Jollow et al. 1974; Hassan et al. 2025; Elsawy et al. 2021). Nevertheless, the present study is subject to certain limitations, most notably the reliance on a single animal model system with male gender only, which may not fully capture human biological complexity. Consequently, further investigation is warranted to define the specific molecular mechanisms and signalling cascades that drive these effects, providing a clearer roadmap for clinical translation. Despite these limitations, this research paves the way for innovative drug design and industrial implementation, offering a more environmentally conscious approach to relevant manufacturing sectors.

## Conclusion

This study provides evidence that naringenin offers significant protection against potassium bromate-induced nephrotoxicity through its multifaceted antioxidant, anti-inflammatory, and cytoprotective properties. These findings support the potential therapeutic application of naringenin in preventing and treating diseases, involving oxidative stress-related nephrotoxicity or compromised renal function.

## Data Availability

All source data for this work (or generated in this study) are available upon reasonable request.
